# Causes and treatment of temporomandibular luxation—a retrospective analysis of 260 patients

**DOI:** 10.1007/s00784-023-05024-z

**Published:** 2023-04-29

**Authors:** Reetta Tarhio, Miika Toivari, Johanna Snäll, Johanna Uittamo

**Affiliations:** Department of Oral and Maxillofacial Diseases, University of Helsinki and Helsinki University Hospital, Helsinki, Finland

**Keywords:** Temporomandibular luxation, Neurological condition, Geriatric age, Oral and maxillofacial surgery

## Abstract

**Objectives:**

We aimed to clarify the etiology, diagnostic process, and treatment of temporomandibular joint (TMJ) luxation, as the standard care is mainly based on case-reports and systematic studies are lacking. The hypotheses were that luxation occurs spontaneously, recurrence manifests particularly among geriatric patients, and surgery is needed infrequently.

**Patients and materials:**

A retrospective study of TMJ luxation patients (*n* = 260) from 2007 to 2020 was designed and implemented. The primary outcome was type of TMJ luxation (i.e., recurrent or non-recurrent), and secondary outcomes were the need for and type of surgical intervention. Predictor variables comprised age, sex, presence of neurological condition, and mechanism of luxation. Administered treatment and clinical outcomes were recorded.

**Results:**

Of luxation, 61.9% was recurrent and 40.0% due to spontaneous cause. Only 1.9% of patients underwent surgical intervention. The presence of neurological condition caused a 1.34-fold risk for recurrence of luxation and general condition a 1.57-fold risk.

**Conclusions:**

TMJ luxation is often recurrent, bilateral, and spontaneous. Recurrent luxation is associated with geriatric and neurological conditions, and in this group recurrent TMJ luxation predicted death.

**Clinical relevance:**

Our findings contribute to more effective diagnostics and treatment of TMJ luxation patients. We show that there is a need to standardize diagnostic measures and treatment patterns. Moreover, collaboration with other specialities, especially neurology and geriatrics, is important.

## Introduction

Luxation of the temporomandibular joint (TMJ) may occur if mandibular condyle dislocation takes place anteriorly over the articular tuberculum of the temporal bone [1]. The simultaneous masticatory muscle spasm blocks the relocation of the condyle behind the articular eminence [2]. The condition is often acute and may cause pain or at least discomfort because the normal position of the lower jaw has changed.

Thorough anamnesis and clinical examination are essential for setting the right diagnosis. Radiology can also be useful to ensure diagnosis. Jaw deviation, inability to close the mouth, and a non-palpable condyle on the posterior side of the articular eminence of temporal bone can indicate TMJ luxation [3]. In bilateral luxation, there is no deviation, however, the mandible is in a prognathic position [4].

Although TMJ luxation is a rare condition relative to joint luxations of other parts of the body and its frequency in the emergency department is only 5.3 cases per year [5], for some patients, the condition is recurrent. Significant predisposing factors have not been found [3], although neurological conditions and changes in connective tissue as in TMJ joint capsule have been reported to be related particularly to recurrent TMJ luxation [6].

Treatment of luxation is manual reduction at the emergency stage [7]. For patients with recurrent TMJ luxation, the acute repositioning of TMJ can only be considered a temporary treatment and needs to be followed by preventive measures [2]. Preventive treatments include non-surgical approaches such as intermaxillary fixation or injection of botulinum toxin into jaw muscles or the TMJ [2, 6]. Surgical methods aim to reform the mandibular condyle, the articular eminence [6].

This study investigated the etiology, diagnostics, and treatment of TMJ luxation and particularly the etiological factors leading to recurrent TMJ luxation, as there is a lack of known predictive factors. The hypotheses were that luxation is predominantly spontaneous, surgical intervention is infrequently considered, and recurrent luxation is more common in the elderly.

## Patients and methods

### Study design

A retrospective cohort study at a tertiary trauma center’s oral and maxillofacial surgery emergency unit (Töölö Hospital Emergency Department, Helsinki University Hospital (HUH), Finland) over a 14-year period from 1 January 2007 to 20 October 2020 was conducted. The oral and maxillofacial emergency service is provided by the Department of Oral and Maxillofacial Surgery, HUH, which has a catchment area of more than 1.6 million inhabitants.

Patients with the following ICD diagnoses were identified from an electronic patient management system: (1) S03.4, sprain and strain of the jaw; (2) S03.0, dislocation of the jaw; (3) S03.5, sprain and strain of the joints and ligaments of other and unspecified parts of the head; (4) K07.62, recurrent TMJ dislocation; and (5) K07.69, unspecific TMJ dysfunction.

### Study variables

The primary outcome was type of TMJ luxation (i.e., recurrent or non-recurrent), and secondary outcomes were need for and type of surgical intervention.

The primary predictor variables comprised age, sex, presence of neurological condition, and mechanism of luxation, which was classified as follows: (1) spontaneous; (2) yawning, vomiting, or eating; (3) dentist visit or gastroscopy; (4) injury; (5) medical seizure; and (6) habit or exercise.

Other variables registered were other general condition(s), regular medication used, type of living conditions, and referral unit. Related to TMJ luxation, we recorded clinical parameters, radiographic interventions, need for manual reduction, and aftercare instructions. Mortality during the study period was also recorded.

TMJ luxation was defined as recurrent if the condition had occurred at least twice in the lifetime.

### Data analysis

Descriptive statistics analyses were conducted. Chi-square test was used to evaluate statistical significance between variables, and 2 × 2 table for risk-ratio calculation.

### Ethical considerations

The internal review board of the Head and Neck Center of HUH (Helsinki, Finland) approved the study. Patient consent was not required due to the retrospective nature of the study.

## Results

The electronic patient record search produced a sample of 800 patients whose medical records were manually re-evaluated. Excluded were patients with any clinical condition other than TMJ dislocation, resulting in the inclusion of 260 patients with confirmed TMJ dislocation.

Table 1 presents the descriptive statistics of 260 patients with TMJ luxation. The incidence was slightly higher (58.1%) among women, and the average age of patients was 51.4 years. The two most common causes by far were spontaneous luxation (40.0%) and yawning, vomiting, or eating (36.2%). Of all luxations, 61.9% were recurrent. Neurological (31.2%) and cardiovascular (27.3%) conditions were the most common general illnesses, and the rate of mortality during the study period was 28.1% in the entire population.

**Table 1:** Descriptive statistic of 260 patients with temporomandibular TMJ luxation

	*n* = 260	% of *n*
Sex		
Male	109	41.9
Female	151	58.1
Age (years)		
Average (range)	51.4 (3.0–100.0)	
0–12 years	6	2.3
13–17 years	12	4.6
18–64 years	143	55.0
At least 65 years	99	38.1
Mechanism		
Spontaneous	104	40.0
Yawning, vomiting, or eating	94	36.2
Dentist visit, gastroscopy	28	10.8
Injury	16	6.2
Fall on the ground or from height	8	2.1
Sports	6	2.3
Hit by blunt object	2	0.8
Medical seizure	16	6.2
Habit/exercise	2	0.8
Delay to seek treatment (days)		
< 1 day	215	82.7
1–2 days	26	10.0
3–7 days	13	5.0
> 7 days	6	2.3
Intoxication (alcohol)		
Yes	24	9.2
Recurrent TMJ luxation		
Yes	161	61.9
Referral quarter		
General practitioner	157	60.4
Dentist	48	18.5
Long-term ward/nursing home	2	0.8
No admittance	53	20.4
General conditions		
Neurological conditions	81	31.2
Cardiovascular diseases	71	27.3
Endocrinological conditions	27	10.4
Musculoskeletal conditions	27	10.4
Respiratory diseases	26	10.0
Psychiatric conditions	23	8.8
Malignancy (any during life)	13	5.0
Developmental disorder	9	3.5
Liver diseases	1	0.4
Living (known for *n* = 90)		
Supported home	42	16.2
Home	32	12.3
Institutional	16	6.2
Mortality within study period		
Yes	73	28.1

Abbreviation: *yrs*, years; *d*, days; *recurrent luxation*, patient having at least two TMJ dislocation during life

Table 2 presents status, radiography, and intervention under primary evaluation for TMJ patients. Of cases, 57.3% were bilateral. Manual reduction was needed for 91.9% of patients and was successful for 69.2% without any medication. Intravenous relaxants and sedation were the most common medications required. Surgical treatment was planned for 15 patients but was cancelled for patients` anesthesiologic contraindications for general anesthesia, and five patients perished before the planned surgery. Eminectomy was carried out for five patients (1.9%).

**Table 2:** Status, radiography, and intervention in 260 patients under primary evaluation for temporomandibular luxation

	*n* = 260	% of *n*
Unable to close mouth		
Yes	245	94.2
Side of luxation		
Bilateral	149	57.3
Unilateral	81	31.2
Un-registered	30	11.5
Radiography		
Panoramatomography	55	21.2
CT of facial bones	19	7.3
Towne projection (mandible)	7	2.7
Plain radiography of TMJ	4	1.5
Manual reduction attempt		
Yes	239	91.9
No	21	8.1
Spontaneous reduction without intervention	13	5.0
Spontaneous relocation after NSAID, relaxant (p.o. or i.v.) or	5	1.9
Local anesthetic		
Relocation successful by first aid or referral unit	3	1.2
Medication for (manual) reduction		
None	180	69.2
Yes	59	22.6
Relaxation, i.v. sedation	33	12.7
General anesthesia	21	8.1
Local anesthetic injection to TMJ	5	1.9
Aftercare		
Compression hood/jaw bandage	144	55.4
Restriction of mouth opening	143	55.0
Soft diet (up to 2–3 weeks)	121	46.5
NSAID-medication cure	78	30.0
Stabilization splint (“nightguard”)	19	7.3
Botulinum toxin injection to masticatory muscles	3	1.2
Cold-package	5	1.9
Mandibular movement exercise	7	2.7
Muscle relaxant	3	1.2
Surgical treatment		
Considered but not needed	15	5.8
Yes	5	1.9

Abbreviations: *TMJ*, temporomandibular joint; *CT*, computed tomography;* i.v.*, intravenous; *p.o.*, per oral

Table 3 presents the comparison of 99 patients with non-recurrent TMJ luxation and 161 patients with reported or clinically confirmed recurrent TMJ luxation under primary evaluation. Patients aged at least 65 years were more common among the recurrent (63.6%) than non-recurrent (36.4%) group, although the difference was not significant. By contrast, the difference in relation to cause of luxation between the groups was significant (*P* = 0.003). Spontaneous luxation was by far more common among the recurrent group (71.2%), whereas injury caused the luxation among non-recurrent patients (81.2%). Dental panoramic radiograph was the predominant radiological intervention for both the recurrent (50.9%) and non-recurrent (49.1%) group; however, for patients in these groups, radiological diagnostic tool was only used in 51.8% and 48.2%, respectively (*P* = 0.019). Altogether, 68.5% of recurrent and 31.5% of non-recurrent patients perished during the study period (*P* = 0.173).

**Table 3:** The comparison of 99 patients with non-recurrent and 161 patients with reported or clinically confirmed recurrent TMJ luxation patient under primary evaluation

	Recurrent		Non-recurrent		
	*n* = 161	%	*n* = 99	%	*P* value
Sex					
Female	97	64.2	54	35.8	.365
Male	64	58.7	45	41.3	
Age (years)					
Average (range)	52.9 (4.0–92.0)		49.1 (3.0–100.0)		
0–12 years	2	33.3	4	66.7	.393
13–17 years	6	50.0	6	50.0	
18–64 years	90	62.9	53	37.1	
At least 65 years	63	63.6	36	36.4	
General condition					< .001
Present	103	74.6	35	25.4	
Absent	58	47.5	64	52.5	
Neurological condition					.004
Present	60	75.0	20	25.0	
Absent	101	56.1	79	43.9	
Cardiovascular condition					.954
Present	45	61.6	28	38.4	
Absent	116	62.0	71	38.0	
Mechanism					
Spontaneous	74	71.2	30	28.8	.003
Yawning, vomiting, or eating	62	65.9	32	34.1	
Dentist visit, gastroscopy	13	46.4	15	53.6	
Injury	3	18.8	13	81.2	
Fall on the ground or from height	3	37.5	5	62.5	
Sports	0	-	6	100.0	
Hit by blunt object	0	-	2	100.0	
Medical seizure	9	56.3	7	43.7	
Habit/exercise	0	-	2	100.0	
Unable to close mouth					
Yes	156	63.7	89	36.3	< .001
Side of luxation					
Bilateral	93	62.4	56	37.6	.969
Unilateral	50	61.7	31	38.3	
Un-registered	18	60.0	12	40.0	
Radiography					
No	117	66.9	58	33.1	.019
Yes	44	51.8	41	48.2	
Panoramatomography	28	50.9	27	49.1	
CT of facial bones	10	52.6	9	47.4	
Plain radiography of TMJ (termi?)	2	50.0	2	50.0	
Towne projection (mandible)	4	57.1	3	42.9	
Manual reduction attempt					
Yes	151	63.2	88	36.8	.159
No	10	47.6	11	52.4	
Spontaneous reduction without intervention	3	23.1	10	76.9	
Spontaneous relocation after NSAID,	4	80.0	1	20.0	
Relaxant (p.o. or i.v.) or local anesthetic					
Relocation successful by first aid or referral unit	3	100.0	0	-	
Medication for manual reduction					
None	119	66.1	61	33.9	.096
Yes	32	54.2	27	45.8	
Relaxation, i.v. sedation	17	51.5	16	48.5	
General anesthesia	13	61.9	8	38.1	
Local anesthetic injection to TMJ	2	40.0	3	60.0	
Aftercare					
Compression hood/jaw bandage	95	66.0	49	34.0	.220
Restriction of mouth opening	87	65.9	45	34.1	
Soft diet (up to 2–3 weeks)	77	63.6	44	36.4	
NSAID-medication cure	43	55.1	35	44.9	
Stabilization splint (“nightguard”)	12	63.2	7	36.8	
Botulinum toxin injection to masticatory muscles*	3	100.0	0	-	
Cold-package	2	40.0	3	60.0	
Mandibular movement exercise	2	28.6	5	71.4	
Muscle relaxant	1	33.3	2	66.6	
Surgical treatment					
Considered**	12	75.0	4	25.0	.440
Yes***	5	83.3	1	16.7	
Mortality within study period					
Yes	50	68.5	23	31.5	.173
≤ 12 months	30	68.2	14	31.8	
> 1 year	20	69.0	9	31.0	
≤ 12 months when neurological condition present	25	71.4	10	28.6	
Time to death from primary contact (days)					
Average (range)	884 (1–4664)		489 (6–3597)		

Abbreviations: *TMJ*, temporomandibular joint; *CT*, computed tomography;* i.v.*, intravenous

^*^Considered for *n* = 6 injected for *n* = 3

^**^Patient deceased or frequency of luxation diminished

^***^Eminectomy; *P* value, chi-square

Table 4 presents the risk ratio in a 2 × 2 table between the presence of general conditions, neurological condition, geriatric age, sex, mortality, and recurrence of TMJ luxation. The risk for recurrent TMJ luxation was 1.57-fold when any general condition, and 1.34-fold when neurological condition was present, *P* < 0.001 and *P* = 0.003, respectively. Notable was also the higher risk in general (RR 1.15), and within 12 months in patients with neurological condition (RR 1.18), although the difference was not statistically significant.

**Table 4:** The risk ratio with 2 × 2 table for recurrent TMJ dislocation

	General condition present	Neurological condition present	Geriatric age	Female sex	Mortality during study period	Mortality < 12 months	Mortality < 12 months when neurological condition present
	RR (95% CI)	RR (95% CI)	RR (95% CI)	RR (95% CI)	RR (95% CI)	RR (95% CI)	RR (95% CI)
Recurrent	1.57 (1.27–1.94)	1.34 (1.12–1.60)	1.15 (0.86–1.27)	1.09 (0.89–1.33)	1.15 (0.94–1.40)	1.12 (0.89–1.41)	1.18 (0.93–1.49)
Non-recurrent	Ref	Ref	Ref	Ref	Ref	Ref	Ref
*P* value*	< .001	.003	.655	.365	.173	.348	.213

Abbreviations: *RR*, risk ratio; *CI*, confidential interval; *ref*, reference

^*^Chi-square

## Discussion

This study investigated the etiology, diagnostics, and treatment of TMJ luxation and particularly the etiological factors leading to recurrent TMJ luxation, as there is a lack of known predictive factors. Our hypotheses were confirmed. Luxations were most often spontaneous, surgical intervention was infrequently considered, and recurrent luxation was more common among elderly patients.

Our results showed that TMJ luxation is typically bilateral and recurrent. Recurrent TMJ luxation is often associated with neurological and cardiovascular conditions. Also, recurrent TMJ luxation can also be used as a factor to predict death when the interval between luxations becomes shorter.

The average age of the patients was 51.4 years and over half were women. Patients with recurrent TMJ luxation were older than those with non-recurrent TMJ luxation. Other studies have found that mean age of TMJ luxation patients was around 40 years [8, 9]. TMJ luxation was rare in children and adolescents in our data, and most TMJ luxations in children have been shown to be caused by trauma [10].

Typically, TMJ luxation was spontaneous. Yawning, vomiting, and eating were common mechanisms of TMJ luxation. Yawning and other spontaneous events have been mentioned as a common cause in other studies as well [1, 4, 8]. Differences exist in the prevalence of trauma in TMJ luxation. The incidence of TMJ luxation caused by an injury in present study was 6.2% corresponding to the rate of previous studies which has varied from 20% [8] or as high as 60% [4]. These differences could be explained by where the study was conducted, and the definition used for trauma. The current study showed that the risk for recurrent TMJ luxation was significantly higher in patients with any general condition (*P* < 0.001) and particularly if neurological (*P* = 0.003) condition was present. Indeed, changes in muscular tonus in neurological conditions can increase the recurrence of TMJ luxation [1], whereas epilepsy, connective tissue disorders [4], and oromandibular dystonia [11] have been described as risk factors for recurrent TMJ luxations.

The rate of mortality was also registered in present study. Notable is that the frequency of mortality was 2.2 times higher in study (*n* = 50) than in the control (*n* = 23) group, although the difference was not statistically significant between the groups. In 2 × 2 risk analysis, the risk of mortality was 1.12-fold with recurrent luxation and 1.18-fold during 12 months from primary contact if neurological condition was present, difference not being statistically significant. The higher rate, and elevated risk of mortality can be explained by the fact that neurological conditions such as dementia is the seventh most common cause of death worldwide, third most common in European women aged at least 65 years, and particularly one the leading causes in Nordic countries [12, 13].

The most common clinical findings in TMJ luxation were jaw deviation, pain, and inability to close the mouth. Radiography was not often used as a diagnostic method because the diagnosis could be confirmed based on the clinical findings alone. The question then arises of whether differential diagnoses have been sufficiently excluded, i.e., fractures of the mandible or mandibular condyle. The radiographic method used under primary evaluation was panorama tomography, and it was completed in 21% of cases. This is in accord with previous findings stating that imaging is rarely needed in the acute situation, but when performed it demonstrates the condyle’s anterior position in relation to the articular eminence [14]. Radiography has been shown to be useful in patients for differential diagnoses or to provide information for further treatment planning [7]. In addition, imaging is recommended in trauma cases since it is important to differentiate fractures from luxation.

Manual reduction of TMJ luxation was usually possible without medication. However, when medication was needed, the most common choice was intravenous sedation. General anesthesia or local anesthetic injections to the TMJ were rarely used. Local anesthesia has been proposed for use when performing manual reduction since the condition is very painful [15]. The success rate of manual reduction is high when it is carried out immediately after the luxation [7]. One reason why TMJ luxation patients are taken to the emergency room could be the lack of expertise in performing the manual reduction procedure at the referring center. Over half of the referrals came from general practitioners, who could with ease learn the procedure.

Overall, the treatment alternatives focused on non-surgical measures, and surgery was indicated in only a few cases. Patients eligible for surgery had recurrent TMJ luxation, which affected their quality of life. It has also been shown that complications are rare in surgical treatment of TMJ. If complications occur, they are usually infections or damage to adjacent structures. Generally, in TMJ surgery arthroscopy is considered to minimize complications compared with open surgery, which is often the choice in surgical treatment of TMJ luxation [16]. One systematic review of the treatment options for recurrent TMJ luxation summarized that there is no good-quality evidence on which treatments lead to long-term elimination of recurrent luxation. The options included, for example, eminectomy, miniplanting of the articular eminence, and down-fracture of the zygomatic arch. The authors noted that surgeons empirically consider eminectomy as the “gold standard” [17]. It is noteworthy that these data cover patients from one clinic during a certain period, and it is likely that some patients previously received treatment for TMJ luxation at another clinic or hospital. Thus, the number of recurrent TMJ luxation patients might be even higher.

## Strengths and limitations

A strength of our study was the large size of the patient population, as the previous literature has mainly been based on case-reports. The study also revealed a relationship between mortality and recurrent luxation, highlighting the importance of collaboration between specialties when considering surgical treatment.

The weakness of the study was its retrospective nature; a prospective study would have provided information on prosthodontic treatment. Luxations that were treated in other units, e.g., dentist’s offices, were not included in our data. Thus, there are probably more challenging luxations in our university hospital data than in our general population.

## Conclusions

To summarize, we showed that TMJ luxation is often bilateral, recurrent, and more common in women. Most of the luxations are spontaneous, and these are often associated with geriatric age and neurological conditions. Major surgical intervention and their benefits should carefully be evaluated, due to the potentially limited lifetime in geriatric patients with neurological conditions who get recurrent TMJ luxation.

## Data Availability

The data supporting the findings of this study are available on request from the authors.
